# SIRT1-Mediated Protective Effect of *Aralia elata* (Miq.) Seem against High-Glucose-Induced Senescence in Human Umbilical Vein Endothelial Cells

**DOI:** 10.3390/nu11112625

**Published:** 2019-11-02

**Authors:** Gi Dae Kim

**Affiliations:** Department of Food and Nutrition, Kyungnam University, Changwon-si 51767, Korea; gidaekim@kyungnam.ac.kr; Tel.: +82-55-249-2176; Fax: +82-505-999-2104

**Keywords:** *Aralia elata* (Miq.) Seem, senescence, endothelial cells, SIRT1

## Abstract

*Aralia elata* (Miq.) Seem (AS) is widely been for treating many diseases, enhancing energy, and boosting immunity; however, its protective effects against high-glucose (HG)-triggered endothelial dysfunction and the potential underlying mechanisms have not been investigated. In this study, we determined the effect of AS on senescence in human umbilical vein endothelial cells (HUVECs) and elucidated the mechanisms underlying its anti-aging effects. The senescence model of endothelial cells (ECs) was established by culturing HUVECs in media containing HG (30 mM). We found that the proportion of senescent (senescence-associated β-galactosidase+) cells in the HG group was significantly higher than that in the control group; however, this increase was suppressed by AS treatment. Moreover, cell cycle analysis revealed that AS (20 μg/mL) significantly recovered HG-induced cell cycle arrest in ECs, and Western blot revealed that AS prevented HG-induced decreases in silent information regulator 1 (SIRT1) level and endothelial nitric oxide synthase (eNOS) phosphorylation. These results show that AS delayed HG-induced senescence in ECs by modulation of the SIRT1/5′ AMP-activated protein kinase and AKT/eNOS pathways.

## 1. Introduction

Senescence of endothelial cells (ECs) impairs vascular functions, leading to aging of tissues and organs [1]. Additionally, senescence can trigger stimuli, such as reactive oxygen species [2], hyperglycemia [3], inflammatory cytokines [4], and telomere dysfunction [5], and is promoted under high glucose (HG) conditions in vitro [6], which can cause cellular injury by induction of oxidative stress [7], apoptosis [8], and downregulation of endothelial nitric oxide synthase (eNOS) [9]. In particular, senescence-associated β-galactosidase (SA-β-gal) activity in ECs is widely used as a biomarker for senescence owing to the simplicity of the assay and its apparent specificity for senescent cells [10].

Silent information regulator 1 (SIRT1), a nicotinamide adenine dinucleotide (NAD+)-dependent class III histone deacetylase, reportedly regulates the cell cycle, senescence, apoptosis, and metabolism by interacting with several molecules, including p53 [11]. In ECs, hyperglycemia accelerates aging-like processes via SIRT1 downregulation [12], and signal molecules, such as 5′ AMP-activated protein kinase (AMPK) involved in the energy sensing pathways, are associated with EC senescence [13]. Additionally, endothelial mitochondrial oxidative stress is implicated in senescent vascular events, and AMPK plays a defensive role in this stress during senescence [14]. The phosphoinositide-3-kinase (PI3K)/AKT signaling pathway is crucial in regulating endothelial function and injury [15]. AKT, the downstream effector of PI3K, encourages cell survival in response to various causes of cell death by mediating EC survival and inducing the production of nitric oxide (NO) by activating eNOS [16].

*Aralia elata* (Miq.) Seem (AS), a small tree or shrub belonging to the *Araliaceae* genus, is a well-known Chinese herbal medicine widely cultivated in northeastern China, Japan, and Korea [17]. Previous studies showed that triterpenoid saponins from AS leaves exert antitumor effects [18], and the bark and roots of AS have been used as tonics and diuretics in the treatment of the common cold, gastric ulcer, diabetes, neurasthenia, hepatitis, and inflammatory diseases in Chinese folk medicine [19]. Furthermore, AS exhibits a series of pharmacological functions, including anti-inflammatory, antioxidant, hypolipidemic, and antidiabetic properties [20,21]. Despite the development and application of AS, few studies have focused on it in preventing EC senescence. Therefore, in this study, we established an in vitro HG-induced senescence model using human umbilical vein endothelial cells (HUVECs) and investigated the effect of AS on inhibiting senescence in order to elucidate the underlying mechanisms.

## 2. Materials and Methods

### 2.1. Materials and Reagents

AS was obtained from Dr. Park (Kyungnam University), and AS extraction, separation, and quality control were performed as described previously [22]. The compound was dissolved in 100% dimethyl sulfoxide (DMSO; Sigma-Aldrich, St. Louis, MO, USA), and a 50 mg/mL stock solution was prepared and stored in small aliquots at −20 °C until needed. 3-(4,5-Dimethylthiazol-2-yl)-2,5-diphenyltetrazolium bromide (MTT), gelatin, and mitomycin C were purchased from Sigma-Aldrich. A SA-β-gal kit (ab65351) was purchased from Abcam Inc. (Cambridge, MA, UK). Horseradish peroxidase-conjugated anti-mouse (GTX213111-01) and anti-rabbit (GTX213110-01) antibodies were purchased from GeneTex Inc. (Irvine, CA, USA). An NO assay kit (#EMSNO) was purchased from Thermo Fisher Scientific (Vienna, Austria). Phosphorylated (p)-p38 (#9216), p-eNOS (#9570), p53 (#9282), p-AMPK (#2537), p-AKT (#9271), and SIRT1 (#9475) antibodies were purchased from Cell Signaling Technology (Danvers, MA, USA). β-actin (sc-47778), p-extracellular-signal regulated kinase (ERK) (sc-7383), cdc2 (sc-54), and cyclin B1 (sc-245) antibodies were purchased from Santa Cruz Biotechnology (Dallas, TX, USA).

### 2.2. EC Culture

HUVECs were obtained from ATCC (Manassas, VA, USA) and cultured in endothelial growth medium-2 (EGM-2; Lonza, Walkersville, MD, USA) supplemented with 10% fetal bovine serum (FBS) at 37 °C in a 5% CO_2_ atmosphere. ECs at passages three through five were used in the experiments [23]. We used the commercially available EGM-2 MV microvascular EGM-2 bullet kit [24]. ECs were cultured in EGM-2 (control), low-glucose (LG; 6 mM), and HG (30 mM) medium with or without AS at different concentrations (5−20 μg/mL) for 48 h at 37 °C in a 5 % CO_2_ atmosphere.

### 2.3. Cell-Viability Assay

The effect of AS on cell viability was calculated as percentages relative to the solvent-treated control. The experimental process followed a previously described method [23]. Briefly, ECs (5 × 10^3^ cells/well) were seeded onto a 96-well plate with EGM-2 medium supplemented with 10% FBS. At confluence, the culture medium was removed, and the cells were rinsed twice with phosphate-buffered saline (PBS) and then incubated with serum-free medium for 12 h. Following serum starvation, the cells were cultured in fresh 2% FBS containing various concentrations of AS at 37 °C for 72 h and then treated with MTT solution for 4 h. The resulting formazan deposits were dissolved using DMSO, and the absorbance was detected at 570 nm using the VersaMax ELISA microplate reader (Molecular Devices, Sunnyvale, CA, USA).

### 2.4. Scratch-Wound Migration Assay

Experiments were conducted according to previously described methods [25]. Briefly, ECs were cultured until confluence in six-well plates pre-coated with 0.1% gelatin and then incubated with 10 mg/mL mitomycin C at 37 °C in a 5% CO_2_ atmosphere for 2 h to inactivate the ECs. EC monolayers were wounded by scratching with a 0.2 mL pipette tip, followed by addition of fresh medium containing various concentrations of AS. Images were captured using an inverted phase-contrast light microscope (Olympus Optical Co. Ltd., Tokyo, Japan) at the point of full migration. The migrated cells in three randomly selected fields were counted using an optical microscope at 200× magnification and quantified by manual counting (DMC advanced program), with inhibited migration calculated as a percentage relative to the control.

### 2.5. SA-β-gal Staining

The SA-β-gal assay was performed using the β-gal kit (Abcam) to determine the number of senescent cells according to manufacturer instructions. ECs were fixed for 5 min in β-gal fixative (2% formaldehyde and 0.2% glutaraldehyde in PBS), washed with PBS, and stained using the β-gal fixative solution (1 mg/mL 5-bromo-4-chloro-3-indolyl-beta-D-galactopyranoside in 5 mM potassium ferricyanide, 5 mM potassium ferrocyanide, and 2 mM MgCl_2_ in PBS) at 37 °C until β-gal staining was visible in either the experimental or control plate. Cells were washed in PBS, and SA-β-gal positive cells (blue staining) were observed by microscopy, with >500 cells counted in three independent fields.

### 2.6. Cell Cycle Analysis

To determine the effect of AS on the level of cell cycle arrest, cells were treated with various concentrations of AS for 24 h according to a previously described method [25]. Briefly, cells were harvested (trypsinization and centrifugation) and fixed with 70% ethanol overnight at 4 °C. The cells were then washed, stained with 50 μg/mL of propidium iodide and 50 μg/mL of RNase A for 1 h in the dark, and then subjected to flow cytometric analysis using the FACSCalibur flow cytometer (Becton Dickinson, San Jose, CA, USA). Events were evaluated for each sample, and cell cycle distribution was analyzed using Cell Quest software (Becton Dickinson). The percentage of cells in the G0/G1, S, and G2/M phases were determined. All experiments were performed in triplicate.

### 2.7. Measurement of NO Production

NO concentrations in supernatants from EC cultures were determined by detecting the concentration of nitrite, the stable product of NO, using an NO assay kit (Thermo Fisher Scientific) according to manufacturer instructions. The optical densities at 560 nm were determined using a microplate reader (Molecular Devices), and NO concentrations were calculated using the calibration curve. Three independent experiments were performed.

### 2.8. Western Blot Analysis

ECs were treated with AS for 24 h, and Western blot was performed according to a previously described method [25]. Briefly, ECs were harvested and lysed in protein-extraction solution (Intron Biotechnology, Inc., Kyunggi, Korea) containing protease inhibitors and phosphatase inhibitors for 10 min at 4 °C. The total protein concentration in the supernatants was measured using the Bradford assay [11]. After heating at 95 °C for 5 min, total protein samples (30 μg) were subjected to 6% to 15% sodium dodecyl sulfate polyacrylamide gel electrophoresis. The proteins were transferred onto polyvinylidene fluoride membranes (Millipore, Bedford, MA, USA) at 100 V for 60 min to 100 min. The membranes were incubated with 5% bovine serum albumin in Tris-buffered saline with 0.05% Tween 20 (TBST) for 30 min at room temperature, and then with diluted primary antibodies (1:200–1:1000) in 5% BSA in TBST overnight at 4 °C. The membranes were washed three times with TBST and incubated with the corresponding secondary antibodies. Protein bands were detected using an enhanced chemiluminescence detection kit (Intron Biotechnology, Inc.) and a LAS-1000 Imager (Fuji Film Corp., Tokyo, Japan). Semi-quantitative analysis of densitometry results was performed using Image J software (National Institutes of Health, Bethesda, MD, USA).

### 2.9. Statistical Analysis

The results are expressed as mean ± standard deviation (SD), with statistical significance determined using one-way analysis of variance. Post hoc comparisons between groups were performed by a Bonferroni multiple comparison test. Calculations were performed using SPSS for Windows (v.23.0; IBM Corp., Armonk, NY, USA), and *p* < 0.05 was considered statistically significant.

## 3. Results

### 3.1. The Effect of AS on EC Viability

**Figure 1:**
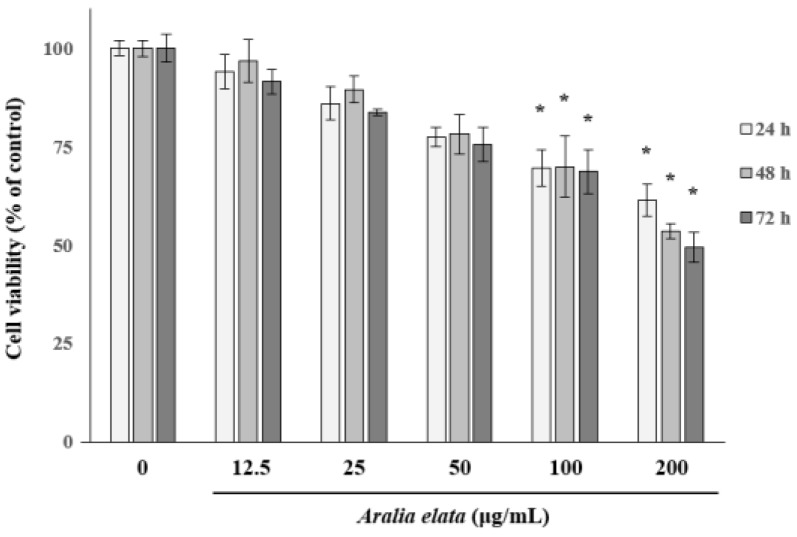
Effects of *Aralia elata* (Miq.) Seem (AS) on HUVEC viability. HUVECs were treated with AS (0–200 μg/mL) for 72 h. Cell viability is expressed as a percentage of viable cells cultured in the absence of AS and expressed as the mean ± SD. * *p* < 0.05.

We first determined the non-cytotoxic concentration of AS by analyzing the EC viability following treatment with different concentrations of AS (0–200 μg/mL) for 72 h. AS concentrations >100 μg/mL significantly (*p* < 0.05) decreased EC viability (Figure 1); therefore, AS concentrations ≤25 μg/mL were used in subsequent experiments to avoid EC cytotoxicity.

### 3.2. Activation of EC Migration by AS

**Figure 2:**
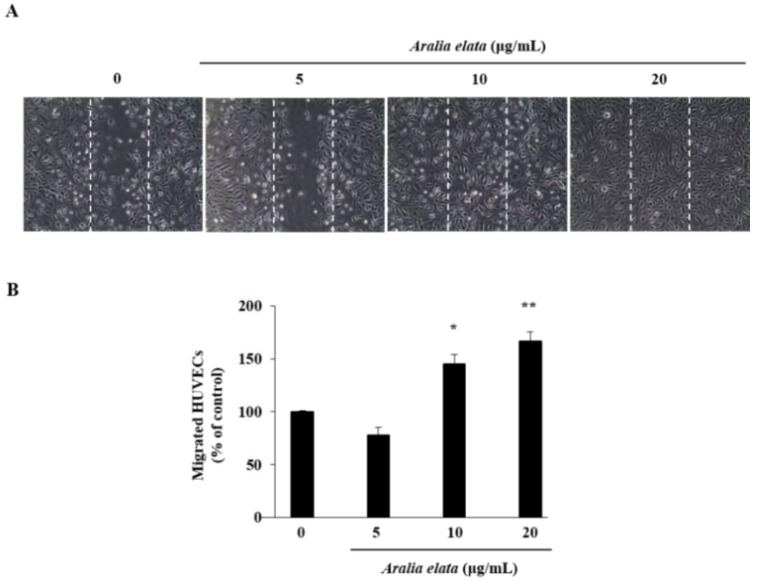
Effects of AS on HUVEC migration. Cells were grown to confluence in six-well plates, wounded, and treated with the indicated concentrations of AS. (**A**) Representative images show cell migration in control and AS-treated HUVECs. (**B**) Cell migration expressed as a percentage of that observed in control cells. Data represent the mean ± SD. * *p* < 0.05, ** *p* < 0.01.

**Figure 3:**
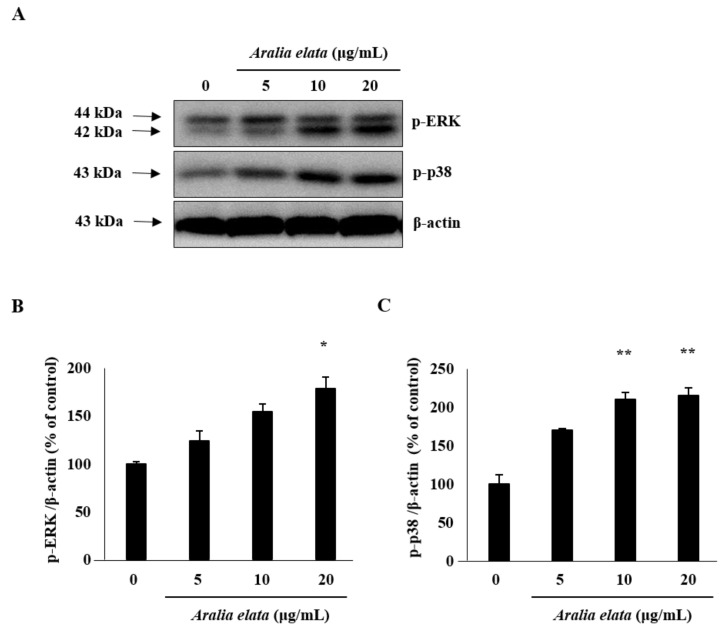
ERK and p38 activation by AS in HUVECs. Cells were incubated with different concentrations of AS (0–20 μg/mL) for 24 h, lysed, and analyzed by Western blot. AS treatment for 24 h increased phosphorylated ERK and p38 levels in HUVECs. (**A**) Representative blots. (**B**,**C**) Quantitative analysis (*n* = 3) of both p-ERK/β-actin and p-p38/β-actin. * *p* < 0.05, ** *p* < 0.01.

The effect of AS on EC migration was confirmed by wound-healing assays in vitro. AS (20 μg/mL) treatment increased EC migration relative to that observed in the control group (*p* < 0.01) (Figure 2A,B). Additionally, Western blot revealed that AS (0–20 μg/mL) induced activation of mitogen-activated protein kinase (MAPK) signaling molecules, including extracellular-signal regulated kinase (ERK) and p38 (Figure 3A), according to dose-dependent increases in p-ERK and p-p38 levels in ECs, with those treated with 20 μg/mL showing significantly higher levels than those in the control group (*p* < 0.05 and *p* < 0.01, respectively) (Figure 3B,C).

### 3.3. Inhibition of HG-Induced EC Senescence by AS

**Figure 4:**
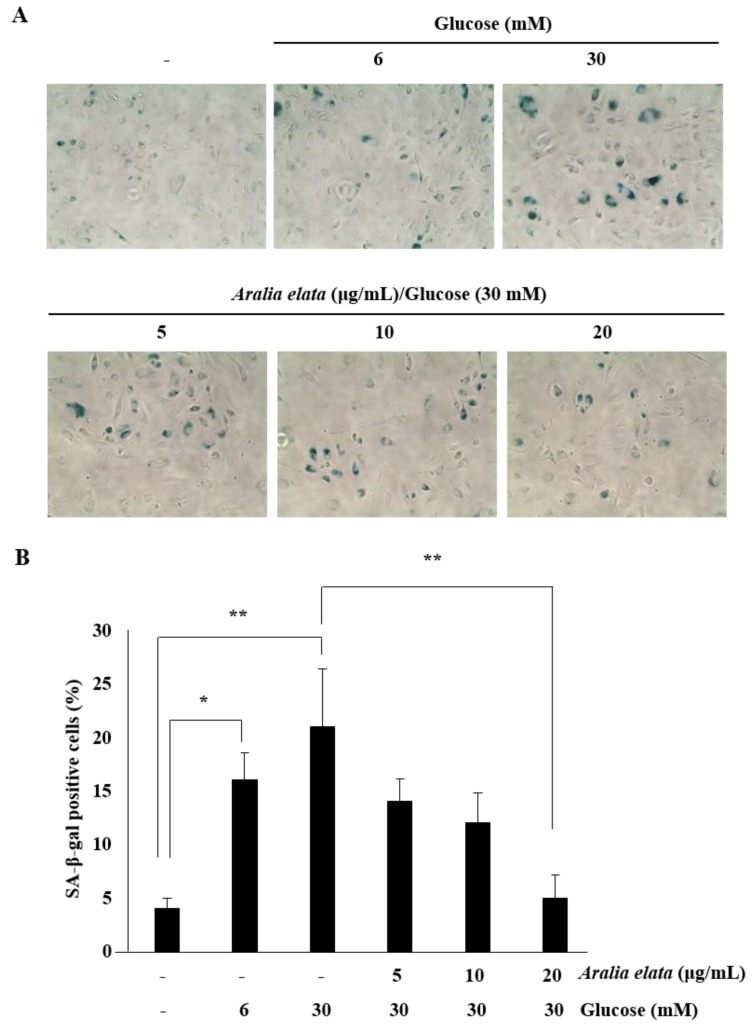
Effects of AS on the senescence of HUVECs cultured with HG medium. (**A**) Representative images of SA-β-gal staining (blue) of HUVECs treated with media alone, media containing HG, or media containing HG and AS (0–20 μg/mL) for 48 h. (**B**) Percentages of SA-β-gal positive cells in the different groups. SA-β-gal positive cells in treatment groups were calculated as a percentage of those in the control group. * *p* < 0.05; ** *p* < 0.01 vs. control group.

Because SA-β-gal is a biomarker of EC senescence [26], we investigated whether transient HG could induce persistent EC senescence, thereby establishing a cellular model for senescence. Here, ECs were subcultured in media containing no glucose, LG (6 mM), or HG (30 mM), and cellular senescence was determined using the SA-β-gal assay. Consistent with a previous study [27], the percentage of senescent ECs increased significantly at 48 h after HG treatment (Figure 4A), with the proportion of SA-β-gal positive cells in the HG group being significantly higher than that in the control group (21.0% ± 5.4% vs. 4.0% ± 1.1% in control cells; *p* < 0.01) (Figure 4B). However, the presence of AS during HG treatment prevented senescence, as the proportion of SA-β-gal positive cells among those treated with HG and 20 μg/mL AS was significantly lower than that in cells treated with HG alone (5.0% ± 2.1% vs. 21.0% ± 5.4% in HG, *p* < 0.01). Moreover, AS suppressed HG-induced senescence in a concentration-dependent manner (5–20 μg/mL) (Figure 4). These results suggested that AS protected ECs from HG-induced damage.

**Figure 5:**
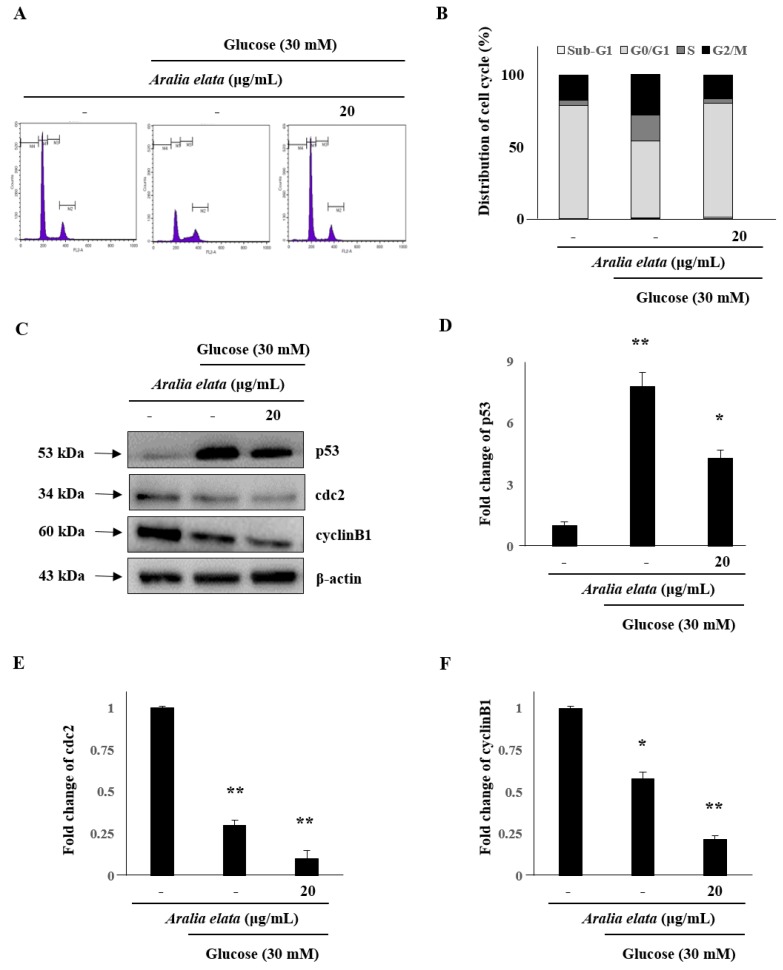
Effect of AS on cell cycle distribution in HUVECs. (**A**) Representative fluorescence-associated cell sorting plots describing cell cycle phases. (**B**) Percentage of cells in different cell cycle phases after HG and AS treatment. Data represent the mean ± SD of five independent experiments. (**C**) Western blot analysis of p53, cdc2, and cyclin B1 levels. (**D**–**F**) Quantification of p53, cdc2, and cyclinB1 levels relative to β-actin. * *p* < 0.05; ** *p* < 0.01 vs. the control group.

The cell cycle is a critical process, with cell cycle arrest exhibited by senescent cells. Therefore, we analyzed the effect of 24 h treatment with AS on the number of cells in different cell cycle phases. The proportion of cells in the G2/M phase in the HG-treated group (27.92%) was higher than that in the control group (17.65%); however, co-treatment with AS (20 μg/mL) prevented HG-related effects and reduced the proportion of cells in the G2/M phase to 16.89% (Figure 5A,B). Furthermore, compared with control cells, HG-treated cells showed significantly increased p53 levels (*p* < 0.01), whereas these levels in cells treated with both AS and HG were considerably lower than those in cells treated with HG alone (Figure 5C,D). Additionally, protein levels of cdc2 and cyclinB1 decreased significantly following AS (Figure 5E,F). On the basis of these results, 24 h was selected for further analyses to elucidate the mechanism underlying AS-mediated reductions in HG-induced toxicity.

### 3.4. Activation of SIRT/AMPK and AKT/eNOS Expression by AS

**Figure 6:**
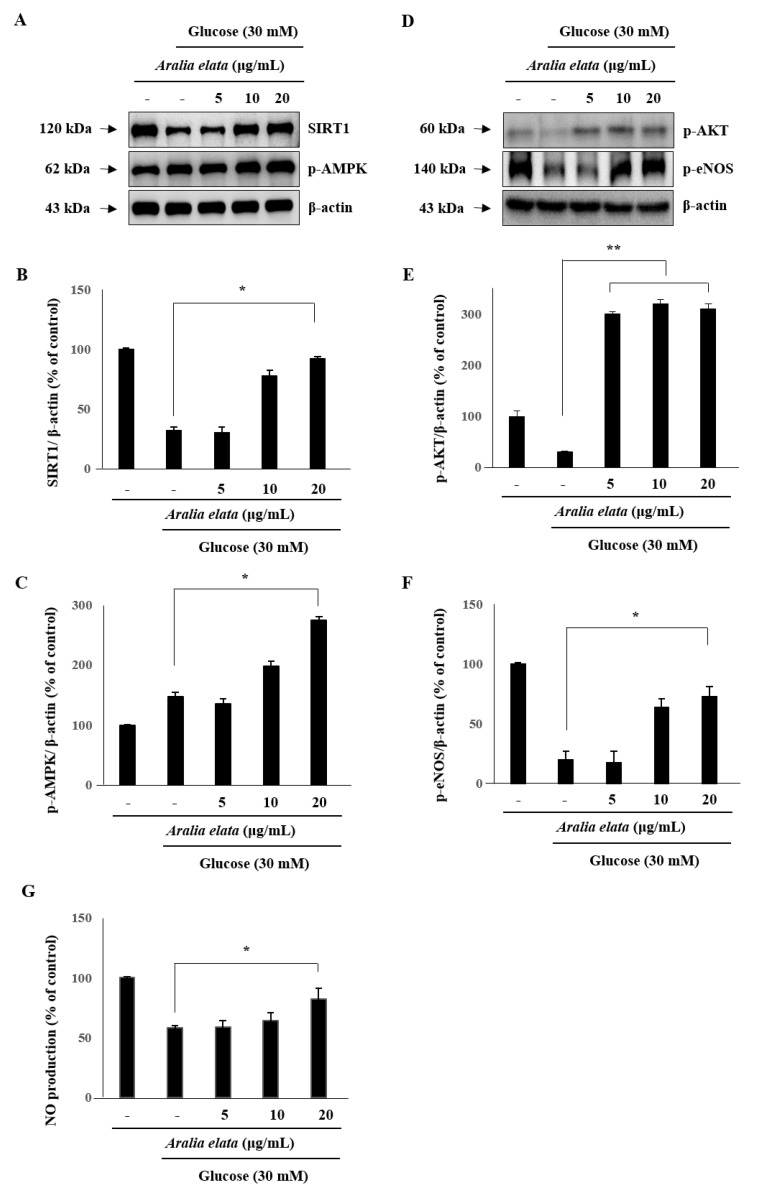
Effect of AS on SIRT/AMPK and AKT/eNOS signaling in HUVECs. Cells were cultured in media alone, media containing HG, or media containing HG and 20 μg/mL AS for 24 h, and then subjected to Western blot analysis and NO production assays. (**A**–**C**) SIRT1 and p-AMPK levels and (**D**–**F**) p-AKT and p-eNOS levels relative to β-actin. (**G**) NO production in the different cell groups. * *p* < 0.05; ** *p* < 0.01 vs. the HG group.

We then analyzed SIRT1 levels in ECs to investigate whether AS regulates EC senescence via a SIRT1-mediated pathway. Western blot indicated that SIRT1 levels decreased significantly after treatment with HG (30 mM), and that this decrease was significantly rescued by treatment with AS (*p* < 0.05) (Figure 6A–C). Furthermore, p-AMPK levels in ECs were significantly increased by AS treatment, suggesting that AS activated the SIRT1/AMPK pathway to protect ECs against HG-induced injury.

We then evaluated AKT and eNOS activation in order to assess whether the protective effect of AS is associated with the AKT/eNOS pathway. HG decreased p-eNOS levels in ECs; however, compared with the HG group, AS treatment increased levels of p-AKT and p-eNOS (*p* < 0.01 and *p* < 0.05, respectively) (Figure 6D–F). Moreover, HG treatment reduced NO production in ECs, which was significantly recovered following AS treatment (*p* < 0.05) (Figure 6G). These results suggested that AS activates the AKT/eNOS pathway by increasing AKT and eNOS phosphorylation.

## 4. Discussion

Cellular senescence is a process characterized by modulations in cell function, morphology, division, and gene expression [28], with EC senescence and the consequent reductions in their proliferative and migration associated with aging. In this study, we established an in vitro EC-based HG-induced senescence model to investigate the protective role of AS in EC senescence, revealing that AS delayed HG-induced EC senescence.

Several studies reported the involvement of MAPKs (JNK, ERK, and p38) in growth, migration, and functional alterations in cancer and ECs. MAPKs are important for cell differentiation, proliferation, and apoptosis, and ERK is involved in cell proliferation [29,30]. In the present study, AS treatment increased EC migration, accompanied by significant increases in p-p38 and p-ERK levels in ECs (Figure 2 and Figure 3).

SA-β-gal is widely used as a marker of cellular senescence [26]. In the present study, AS treatment reduced HG-induced increases in SA-β-gal positive cells, indicating that SA treatment attenuated HG-induced EC senescence. ECs undergoing premature senescence are protected by SIRT1, a mammalian homologue of the seven sirtuins [31], which regulates inflammation and anti-aging in ECs and represents a key regulator of cell defense, metabolism, and survival [32]. A previous study reported that HG decreases the EC number by reducing SIRT1 levels and enzyme activity [33]. Notably, we found that AS treatment upregulated SIRT1 levels in HG-induced ECs. Another study reported that SIRT1 is closely associated with the acetylation status of p53 [34]. In the present study, we found that HG significantly increased p53 levels relative to those in the control group (Figure 5), possibly resulting in the increased number of SA-β-gal positive cells HG-treated ECs (Figure 4 and Figure 5). These findings suggested that sustained SIRT1 downregulation and p53 upregulation might be associated with EC senescence.

AMPK represents an important metabolic switch associated with modulating cellular and whole-body metabolism. According to previous studies, AMPK is essential for maintaining vascular endothelial health and exerts a regulatory effect on endothelial function and vascular structure [35]. As an AMPK regulator, SIRT1 exhibits NAD^+^-dependent deacetylase activity; however, AMPK phosphorylation also regulates SIRT1 activity [36]. Therefore, upregulating SIRT1/AMPK activity might represent an effective strategy for restraining glucose-induced endothelial dysfunction [37]. In the present study, we found that AS treatment promoted AMPK activity against HG-induced EC senescence (Figure 6). Additionally, SIRT1 modulated NO synthesis by controlling eNOS levels in ECs, which agreed with a previous study reporting SIRT1-mediated enhancement of NO production by deacetylating eNOS [38]. In the present study, we observed that HG significantly decreased eNOS phosphorylation and NO production, whereas co-treatment with AS rescued p-eNOS levels and NO production. Furthermore, a recent report indicated that PI3K/AKT signaling is associated with eNOS activity [18], with AKT activation triggering cell proliferation and survival via phosphorylation of various substrates to suppress the levels of pro-apoptotic proteins [39]. Moreover, eNOS, as a key enzyme involved in regulating endothelial NO production, is regulated by AKT [40]. These findings suggest AS as a regulator of AKT/eNOS signaling to prevent EC damage and exert a protective effect against HG-induced senescence.

## 5. Conclusions

This study demonstrated the efficacy of AS for increasing EC migration via elevated phosphorylation of p38 and ERK, as well reducing HG-induced EC senescence. In conclusion, we suggest that AS protects endothelial cells from HG-induced cytotoxicity by upregulating the SIRT1/AMPK and AKT/eNOS signaling pathways, and might act as a potential therapeutic candidate for the treatment of endothelial cell senescence.
